# Is there an increase over time in the complexity of teacher questions and student responses in case-based clinical seminars? A cross-sectional video study

**DOI:** 10.1186/s12909-022-03944-0

**Published:** 2022-12-15

**Authors:** Martin Gartmeier, Alexander Hapfelmeier, Marc Grünewald, Janina Häusler, Theresa Pfurtscheller, Tina Seidel, Pascal Berberat

**Affiliations:** 1 Technical University of Munich, School of Medicine, TUM Medical Education Center, Munich, Germany; 2 Institute of General Practice and Health Services Research, Techincal University of Munich, TUM School of Medicine, Munich, Germany; 3 Department of Ophthalmology, Technical University of Munich, TUM School of Medicine, Munich, Germany; 4 Technical University of Munich, TUM Graduate School, Munich, Germany; 5 Friedl Schöller Endowed Chair for Educational Psychology, Technical University of Munich, TUM School of Education, Munich, Germany

**Keywords:** Teacher questions, Case-based learning, Video study, Student elaboration

## Abstract

**Background:**

Case-based group discussions (CBGD) are a specific, interaction-focused format dedicated to fostering medical students’ skills in applying basic biomedical knowledge to patient cases. Existing conceptions of CBGD suggest that a gradient towards increased opportunities for students to make elaborative verbal contributions is an important element of such seminars. To verify this assumption, we investigate empirically if clinical teachers progress from more basic, knowledge-oriented questions towards more advanced, elaboration-oriented questions in such seminars.

**Methods:**

We videotaped 21 different clinical teachers and 398 medical students in 32 CBGD-seminars on surgery and internal medicine. We coded closed-reproductive and open-elaborative teacher questions as well as reproductive and elaborative student responses to these questions. Inter-rater reliability was satisfactory. To determine trends regarding the teacher questions / student responses, we compared eight time-segments of equal duration per seminar.

**Results:**

Overall, clinical teachers asked more closed-reproductive than open-elaborative questions. Students gave more reproductive than elaborative responses. Regarding the frequencies of these forms of teacher questions / student responses, we found no significant differences over time.

**Conclusions:**

Clinical teachers did not deliberately modify the types of questions over time to push students towards more elaborative responses. We conclude that the critical question to which degree promising teaching approaches are actually put into clinical teaching practice should be raised more purposefully in medical education research.

## Background

Case-based pedagogic approaches are essential in the context of medical education [1, 2]. Case-based courses are characterized by the application of basic biomedical knowledge to authentic patient-cases; thereby, clinical reasoning and decision making are performed along key-phases of clinical case management, such as clinical examination, diagnosis and therapy. In the present study, we focus upon case-based group discussions (CBGD), a specific form of case-based learning. In CBGD, a clinical teacher guides students through the phases of clinical case management by asking questions, explaining subject matter and encouraging discussion with and among students [3–5]. The clinical teacher (and not the students, as in other case-based formats) is the primary source of teaching. Still, the goal of CBGD is in line with key aspects of case-based medical education in general, i.e., to bridge the gap between basic biomedical knowledge and clinical work, to invite students to actively contribute to class and, in this way, to prepare them for their role as clinicians.

In the context of CBGD, a challenge for clinical teachers is to adjust the level of subject matter complexity and, specifically, of questions asked to students’ level of knowledge [6, 7]. If a teachers’ questions are too difficult, students will feel overburdened; if questions are too easy to answer, they will become bored. Both situations can lead to students’ feeling frustrated and detached from teaching. Moreover, as CBGD is intended to bridge theory and clinical practice and prepare students for the latter, students should not only demonstrate that they possess relevant factual knowledge, but have opportunities to apply knowledge to clinically relevant problems and decisions and engage in clinical reasoning.

Some empirical studies give insights into the kinds of questions clinical teachers ask students in CBGD [8] and other medical teaching formats [9]. However, these studies do not incorporate process-oriented aspects of teaching [10], specifically whether and how clinical teachers progressively modify their questions over time, for instance regarding their complexity, in the course of a teaching session. Especially the progression from lower to higher teaching complexity and learner autonomy is an established pedagogic principle frequently described in the medical education literature [11]; moreover, it is an essential element of widely accepted theoretical models, e.g. the concepts of entrustable professional activities [12] and learners’ zone of proximal development [13].

The former model suggests that with growing professional experience, young physicians perform increasingly complex clinical tasks more and more autonomously. The CBGD-seminars focused on in our study are supposed to prepare medical students for entry to clinical work environments. Hence, it behooves clinical teachers to apply the idea of progressively increasing degrees of complexity and freedom of argumentation in this context also. The latter model, i.e., learners’ zone of proximal development, represents a more learning-theoretical perspective on this pedagogic strategy: Learning theorists argue that for learning to be effective, the complexity of problems learners are confronted with should be slightly outside of their zone of *actual* development [13]. In this way, learners are not overwhelmed, but are challenged to increase their understanding, e.g., by discovering new ways to apply their knowledge. To put this idea into practice, (clinical) teachers need to identify learners’ zone of actual development (e.g. through less complex, primarily fact-oriented questions) and, from there on, move into learners’ zone of *proximal* development. Applied to CBGD, both approaches suggest that teachers initially pose basic questions (lower difficulty, focus on facts) and progress towards more advanced questions (higher difficulty, demanding transfer), in order to urge medical students to apply knowledge and explore new connections [14].

Whether clinical teachers apply this basic pedagogic strategy in context of CBGD, however, has not yet been empirically investigated. This lack of empirical insight presents a gap in the literature for at least two reasons: First, an existing study from surgical education shows that clinical teachers do not modify questions according to learners pre-existing knowledge [9]. In order to estimate whether this is a more widespread problem in medical education, more evidence from other medical disciplines, phases of medical education and pedagogic contexts is required. Second, empirical educational researchers have argued that analyzing features of dialogue is an essential step towards an in-depth understanding of what makes teaching and learning effective in many pedagogic contexts [15]. In addition, this kind of a base-line analysis of dialogic instructional patterns provides a fruitful basis for designing pedagogic interventions and professional training. To date, we were able to identify only one existing study from medical education that adopts such a perspective with respect to CBGD [8]. We see this as a substantial gap in the literature, given the fact that literally all descriptions of case-based medical education stress that the interaction between students and clinical teachers is one of its key characteristics [1, 16]. Existing research shows that it is difficult for teachers to validly judge their own teaching because they tend to overestimate their performance [17]. We hence argue that a video-analytic methodological approach inspired by research on dialogic teaching will help gain more objective and more detailed insights into CBGD as a form of case-based medical education. On this basis, we address the following research questions: In context of CBGD seminars, 1) Do clinical teachers move from asking more basic, reproductive to more complex, deliberative questions? and, 2) Does the number of deliberative student responses increase over time as compared to reproductive student responses?

## Materials and methods

### Sample and recording procedure

During winter-semester 2016/17, we videotaped 32 case-based clinical seminars (16 in internal medicine and 16 in surgery) taught by 21 different clinical teachers (we filmed two teachers five times, three teachers two times and 16 teachers once). In each seminar, a patient-case from the respective clinical discipline was discussed. The initial didactic idea of the seminars was that a clinical teacher should guide and involve students in analyzing and discussing a specific patient case. The topics covered were initial patient case presentation and physical examination, discussion of initial findings, generation of working hypothesis and differential diagnoses, discussion of diagnostic measures (laboratory, radiology, MRT, EKG, etc.) and interpretation of results, diagnostic and therapeutic consequences, follow-up and case summary. The teachers were also supposed to review basic biomedical knowledge relevant for the case at hand. The clinical teachers came from different hospital wards and it was unclear to what degree they had been familiarized with the pedagogic conception of the CBGD seminars. It is well possible that some clinical teachers had just been handed the relevant instructional materials (e.g. power point slides), but had received only a brief introduction on the didactic approach and content the course. Regarding the modification of questions over time from basic to more complex, no specific instructions were given to the clinical teachers.

On average, the clinical teachers in our sample were 38 years old (*SD* = 6.3) and had worked in their profession for 10 years (*SD* = 6.7) on average. In the present sample, only three teachers were female and 16 were male. On average, 15 students took part in each seminar (*SD* = 2.5, *Min* = 10, *Max* = 20); their mean age was 24 years (*SD* = 3.0) and on average, they were in their 8th semester of medical studies (*SD* = 0.9). All students had been informed about the study in advance. Where students did not agree to be video recorded, they were offered to change into another seminar. In case they did not want to be videotaped when their seminar started, they were offered to be seated outside the angle of the camera. To collect coherent data, we developed a standardized procedure of videotaping the seminars [18] and trained all researchers involved in the study accordingly. The average duration of a seminar was 83 min (*Min* = 62, *Max* = 104, *SD* = 10.9).

### Instruments and variables

To collect demographic information, we distributed a questionnaire to all study participants. All other data analyzed in this study were based upon video-analyses. For this purpose, a hierarchical categorical scheme was created based upon published rating schemes from previous video studies [18, 19]. Here, we focussed upon the prevalence of two combinations of codes describing teacher questions, i.e., closed-reproductive questions and open-elaborative questions. The closed-reproductive questions had very few (or even only one) right answer(s). They were often focused upon basic knowledge which students should be familiar with, e.g. from preclinical or clinical courses. An example of such a question from the video-study is *“Which lab values do you need to look at in order to determine a liver problem?”* This question focuses on information also relevant beyond the specific case discussed in the seminar. Moreover, we focused on open-elaborative questions posed by clinical teachers. Such questions did not require a specific number of correct answers, but allowed for differentiated, deliberative answers. Accordingly, they did not focus upon specific information and respondents needed to apply their knowledge in a reflective and evaluative way. An example of an open-elaborative question from our video study is *“Given the age and background of the patient, which therapy would you recommend?”* Focusing on and differentiating between closed-reproductive and open-elaborative questions allowed us to analyze whether clinical teachers in case-based seminars actually move from posing more basic (i.e., closed-reproductive) questions towards questions which impose higher cognitive demands upon students (i.e., open-elaborative questions). We excluded the other two possible combinations (open-reproductive and closed-reasoning) of these categories from our analyses, mainly because they do not as consistently represent a rather narrow focus on facts and their reproduction vs. an orientation towards open reflection.

Regarding student answers to teacher questions, we also focussed on reproductive and elaborative responses: Reproductive answers are mostly brief and contain very limited information, such as one piece of basic knowledge. More often than not, reproductive responses consisted only of one single word. In contrast, elaborative answers involved more lengthy student contributions that contained considerations relevant for clinical decision making, such as descriptions of cause-effect relationships, weighting of arguments or clinical reasoning processes.

### Coding process

In the following, we describe the coding process with a focus on the variables analyzed in the present study (See [8] for a more comprehensive description). Coding was done in three rounds using the software Mangold Interact [20]. In coding round one, the raters trained using the categorical scheme with videos from a pilot study – which were not part of the main study sample – until very good overall inter-rater reliability (Cohen’s Kappa > 0.80) was achieved. In round two, the main study videos were segmented to determine speakers (clinical teacher vs. student/s) and other surface level aspects (e.g., media use). In round three, further codes were applied to the video material to categorize the content of the verbal contributions, specifically regarding types of teacher questions / student responses. The main study videos were divided between four coders. To ensure good reliability, two videos were analyzed by all four coders, resulting in a substantial [21] to good [22] IRR-value of Cohen’s Kappa = 0.65. The IRR values for the specific variables that were the focus of the present study were 0.59 for teacher questions (21: moderate / 22: moderate) and 0.62 for student responses (21: substantial / 22: good).

### Statistical analyses

The distribution of continuous data is presented by mean, range and standard deviation. Qualitative data is described by absolute and relative frequencies. To analyze frequencies of teacher questions and student responses over time, and to standardize seminars of different length, we used deciles to divide seminars into ten equally spaced time intervals. If a seminar lasted 90 min, for instance, the video was divided into 10 intervals of 9 min each; if a seminar lasted 80 min, each interval was 8 min long. For each seminar and time interval, we then estimated the relative frequency of question / response types and illustrated their distribution across seminars by boxplots. A possible non-linear time trend in the respective median values was assessed by linear regression models with an orthogonal polynomial of degree 2. For each seminar, we excluded the first and the last time interval from all further analyses. This was because all seminars started and ended with clinical teachers clarifying organizational matters, such as whether all participants were present or whether participants needed their signature to confirm seminar attendance, etc. All analyses reported here were carried out using the software R 4.0.3. 

## Results

We were able to identify 511 teacher questions coded as closed-reproductive and 424 open-elaborative teacher questions in our video material. On average, teachers asked 22 (*Min* = 2, *Max* = 95) closed-reproductive and 19 (*Min* = 4, *Max* = 50) open-elaborative questions per seminar. Regarding student responses, we identified 577 reproductive and 434 elaborative student responses. On average, students gave 26 (*Min* = 6, *Max* = 102) reproductive responses and 21 (*Min* = 3, *Max* = 59) elaborative responses per seminar.

**Fig. 1:**
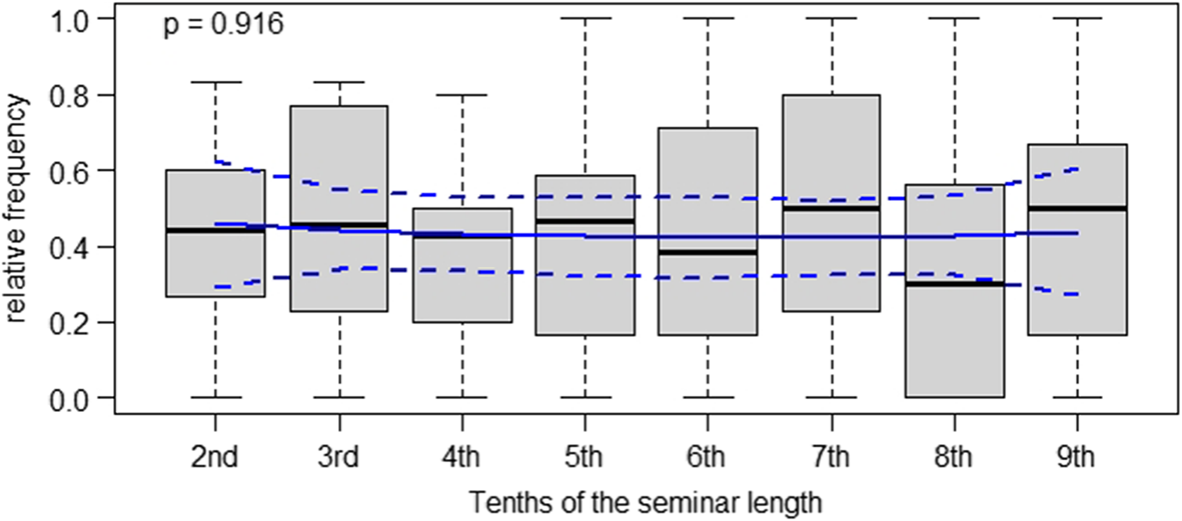
Relative frequencies of closed-reproductive teacher questions in case-based clinical seminars in tenths of the seminar duration

**Fig. 2:**
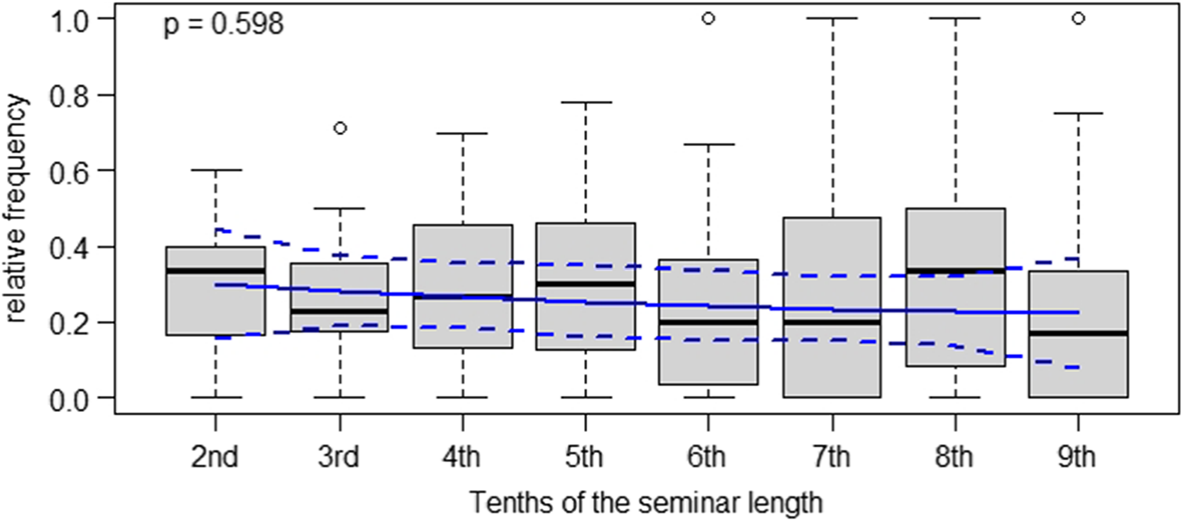
Relative frequencies of open-elaborative teacher questions in case-based clinical seminars in tenths of the seminar duration

**Fig. 3:**
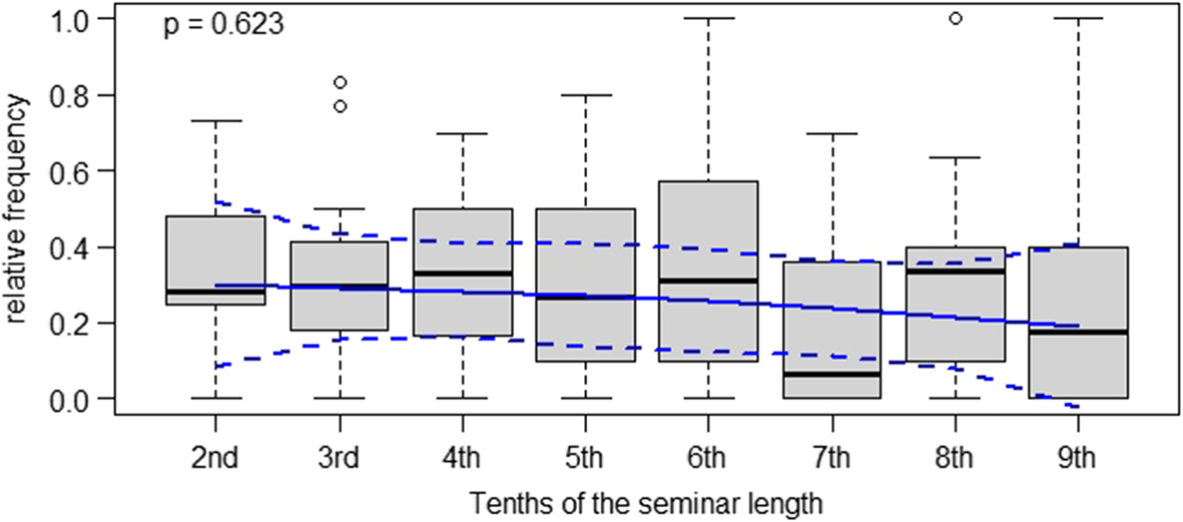
Relative frequencies of reproductive student responses in case-based clinical seminars in tenths of the seminar duration

**Fig. 4:**
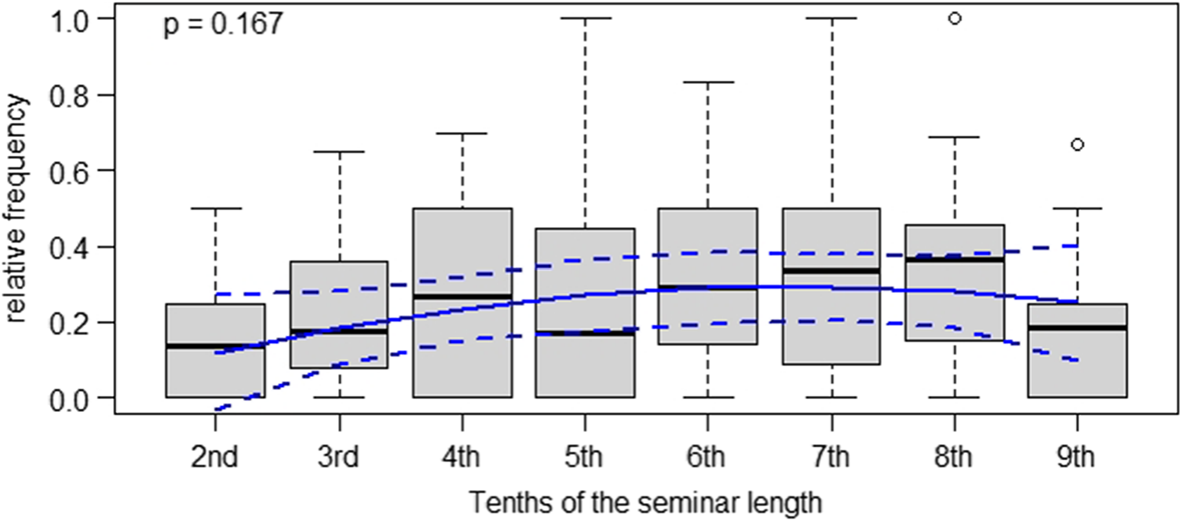
Frequencies of elaborative student responses in case-based clinical seminars in tenths of the seminar duration

In Figs. 1, 2, 3, and 4, we report the frequencies of teacher questions / student responses in case-based seminars over time. Figure 1 shows the frequency of closed-reproductive questions asked by clinical teachers. We observed that clinical teachers posed such questions in a constant fashion during their seminars, with only minor differences between the time intervals (cf. Figure 1). Accordingly, no significant differences between time intervals emerged here.

As is apparent from Fig. 2, clinical teachers posed open-elaborative questions with a lower overall frequency than closed-reproductive questions (cf. Figure 1). However, we did not observe a trend in such questions being posed more frequently towards the end of the case-based clinical seminars. In contrast, the mean values indicate a slowly declining trend regarding such questions though we did not detect statistically significant differences between different time intervals.

Regarding student responses to clinical teacher questions, frequencies of reproductive responses also indicated a slowly declining trend in such statements being made across the duration of clinical seminars. However, no substantial differences between time intervals were found.

Finally, regarding elaborative student responses, we observed a positive trend in over the course of the seminars which, however, was not statistically significant. The overall tendency of these results suggests an increase in students’ ability to elaborate on the clinical management of specific patient cases.

## Discussion

In the present study, we studied case-based group discussions as a form of case-based clinical teaching. Our research questions were whether clinical teachers move from asking closed, fact-oriented questions towards open, elaboration-oriented questions over the course of case-based seminars. Moreover, we investigated how frequencies of reproductive and elaborative responses given by students develop over time across the clinical seminars. These research questions were analyzed based upon video data collected in case-based clinical seminars and analyzed by multiple raters with good reliability. To determine trends regarding the teacher questions / student responses, we compared eight equally long time-segments per seminar. We detected no statistically significant differences regarding any of the variables. However, an increase in elaborative student contributions over time was apparent.

Conceptual descriptions of case-based didactic approaches in medical education stress that these are useful in bridging the gap between theory and practice, teaching clinical reasoning to advanced medical students and helping them acquire clinically relevant problem-solving skills [1, 16]. However, given the results of our study, it is not apparent that the clinical teachers in the seminars managed to meet these goals in their instructional practice. Our data tentatively indicates that students contributed elaborative statements to class with a positive but non-significant trend towards the end of the case-based seminars. At the same time, clinical teachers used a constant rate of closed, reproduction-oriented questions until the final stage of the seminars. Hence, it is not apparent that clinical teachers modified the types of questions they asked to push their students to elaborate, for instance, upon the pros and cons of different therapeutic strategies. As other authors have put it, “reliance on lower level questions diminishes the learner’s need to synthesize and formulate higher level answers” ( [9], p.544). In that sense, our results suggest that the case-based seminars in our study were dominated by an informal, teacher-initiated division of labor between students and clinical teachers: Seemingly, the former were primarily responsible for providing facts, giving keywords and throwing brief ideas into the discussion, while the latter took responsibility for weighing and evaluating these for their relevance in clinical practice. However, as is apparent from Figs. 1, 2, 3, and 4, substantial variance was apparent in our data. This points towards great heterogeneity between the seminars analyzed our study – which, overall, makes it difficult to identify statistically significant effects. For future research as well as for didactic interventions, this suggests that individual differences regarding starting conditions and teaching approaches between different teachers should be more strongly taken into account [23]. Further, a more fundamental question is whether it is legitimate in the first place to expect clinicians teaching case-based courses to “intuitively” modify the difficulty of questions over time *OR* whether such a didactic approach requires specific training. Although our results suggest the latter assumption, we argue that exploratively seeking to identify naturally occurring teaching patterns is still worthwhile. This is because many scholars have theoretically and empirically investigated *intuition* as a resource teachers use in their daily work (e.g., Johansson & Kroksmark, 2010).

In the introduction, we have drawn upon the concepts of entrustable professional activities [12] and learners’ zone of proximal development [13] to substantiate the importance of moving from basic to more advanced questions. Our results suggest that clinical teachers did not deliberately attempt to push towards their students’ zone of proximal development and did not progressively entrust a leading role to students in undertaking the more complex steps in analyzing the case at hand. In most cases, clinical teachers are much better able to analyze patient cases than their students based on their clinical experience and knowledge. However, in the context of case-based teaching, it would be advisable to deliberately “suppress” this ability. Our study suggests that this is a challenging step for clinical teachers.

We hold that our results advance research on case-based didactic approaches in medical education, specifically of CBGD. The results of the present study highlight the fact that researchers should put more emphasis on investigating how teachers and learners interact in CBGD, as well as in other, dialogue-focused formats in medical education [24]. Evidence from school settings shows that productive, learning oriented instructional discourse has measurable positive effects, e.g., regarding learning outcomes [25–27]. With respect to medical education, such effects are just as probable but have rarely been deliberately investigated. Results which underline this conjecture come from studies on *pimping* or *prodding* [28–30]. Through this form of direct questioning of medical students, clinical teachers deliberately attempt to disclose students’ knowledge gaps with the potential danger of humiliating them in front of their peers. The fact that there are dozens of scholarly publications on this issue underscores that dialogic instructional practices – also critical ones! – are essential aspects of medical education. Video-based studies on teacher-student interaction in medical education are hence promising, on the one hand, to more precisely describe and contextualize such phenomena; on the other hand, as a valuable basis for conceptualizing didactic interventions dedicated to improving dialogic teaching practices [31, 32].

Finally, we must address the limitations of the present study and, on this basis, draw conclusions for future research. First, the clinical teachers in our study were not specifically trained or instructed to move from asking more closed towards more open questions. It is possible that targeted didactic measures, such as an initial didactic briefing or the provision of guiding questions with growing degree of openness, would have led clinical teachers to modify their questions in the hypothesized way in the course of the seminars. In our view, this would be a promising perspective for future research. Moreover, we argue that the present study provides some pointers as to how more student-centered and effective interaction-based medical education might be achieved. However, a further limitation of our research approach is that we did not directly analyze student–teacher interaction patterns as they dynamically unfold during teaching. Instead, we counted all clinical teacher / student utterances relating to specific question / response categories and statistically explored their distribution over time. From a research strategy point of view, this is a promising first step; however, our study does not indicate *why* clinical teachers did not pose more open, elaboration-oriented questions. A second point relates to the fact that we empirically investigated CBGD as one specific form of case-based clinical teaching. Other, potentially more widely practiced forms of case-based teaching include elements which give learners a more active role and greater responsibility over prolonged periods of time. Based on the present results, we argue that this is one way to prevent clinical teachers from playing a too dominant role during the more complex, reasoning-oriented phases of case-based seminars.

## Data Availability

All data underlying the reported analyses (except the original video recordings) are available upon request from the authors.
